# A Longitudinal Study of Medicaid Coverage for Tobacco Dependence Treatments in Massachusetts and Associated Decreases in Hospitalizations for Cardiovascular Disease

**DOI:** 10.1371/journal.pmed.1000375

**Published:** 2010-12-07

**Authors:** Thomas Land, Nancy A. Rigotti, Douglas E. Levy, Mark Paskowsky, Donna Warner, Jo-Ann Kwass, LeAnn Wetherell, Lois Keithly

**Affiliations:** 1 Massachusetts Tobacco Cessation and Prevention Program, Boston, Massachusetts, United States of America; 2 Tobacco Research and Treatment Center, General Medicine Division, Department of Medicine, Massachusetts General Hospital and Harvard Medical School, Boston, Massachusetts, United States of America; 3 Mongan Institute for Health Policy, Massachusetts General Hospital and Harvard Medical School, Boston, Massachusetts, United States of America; 4 Office of Medicaid Commonwealth of Massachusetts, Boston, Massachusetts, United States of America; The University of Queensland, Australia

## Abstract

**Background:**

Insurance coverage of tobacco cessation medications increases their use and reduces smoking prevalence in a population. However, uncertainty about the impact of this coverage on health care utilization and costs is a barrier to the broader adoption of this policy, especially by publicly funded state Medicaid insurance programs. Whether a publicly funded tobacco cessation benefit leads to decreased medical claims for tobacco-related diseases has not been studied. We examined the experience of Massachusetts, whose Medicaid program adopted comprehensive coverage of tobacco cessation medications in July 2006. Over 75,000 Medicaid subscribers used the benefit in the first 2.5 years. On the basis of earlier secondary survey work, it was estimated that smoking prevalence declined among subscribers by 10% during this period.

**Methods and Findings:**

Using claims data, we compared the probability of hospitalization prior to use of the tobacco cessation pharmacotherapy benefit with the probability of hospitalization after benefit use among Massachusetts Medicaid beneficiaries, adjusting for demographics, comorbidities, seasonality, influenza cases, and the implementation of the statewide smoke-free air law using generalized estimating equations. Statistically significant annualized declines of 46% (95% confidence interval 2%–70%) and 49% (95% confidence interval 6%–72%) were observed in hospital admissions for acute myocardial infarction and other acute coronary heart disease diagnoses, respectively. There were no significant decreases in hospitalizations rates for respiratory diagnoses or seven other diagnostic groups evaluated.

**Conclusions:**

Among Massachusetts Medicaid subscribers, use of a comprehensive tobacco cessation pharmacotherapy benefit was associated with a significant decrease in claims for hospitalizations for acute myocardial infarction and acute coronary heart disease, but no significant change in hospital claims for other diagnoses. For low-income smokers, removing the barriers to the use of smoking cessation pharmacotherapy has the potential to decrease short-term utilization of hospital services.

*Please see later in the article for the Editors' Summary*

## Abstract 2

Thomas Land and colleagues show that among Massachusetts Medicaid subscribers, use of a comprehensive tobacco cessation pharmacotherapy benefit was followed by a substantial decrease in claims for hospitalizations for acute myocardial infarction and acute coronary heart disease.

## Editors' Summary

**Background:**

Smoking is the leading preventable cause of death in the world. Globally, it is responsible for one in ten deaths among adults. In developed countries, the death toll is even higher—in the USA and the UK, for example, one in five deaths are caused by cigarette smoking. In the USA alone, where a fifth of adults smoke, smoking accounts for more than 400,000 deaths every year; globally, smoking causes 5 million deaths per year. On average, smokers die 14 years earlier than nonsmokers, and half of all long-term smokers will die prematurely because of a smoking-related disease. These diseases include lung cancer, other types of cancer, heart disease, stroke, and lung diseases such as chronic airway obstruction, bronchitis, and emphysema. And, for every smoker who dies from one of these smoking-related diseases, another 20 will develop at least one serious disease because of their addiction to tobacco.

**Why Was This Study Done?:**

About half of US smokers try to quit each year but most of these attempts fail. Many experts believe that counseling and/or treatment with tobacco cessation medications such as nicotine replacement products help smokers to quit. In the USA, where health care is paid for through private or state health insurance, there is some evidence that insurance coverage of tobacco cessation medications increases their use and reduces smoking prevalence. However, smoking cessation treatment is poorly covered by US health insurance programs, largely because of uncertainty about the impact of such coverage on health care costs. It is unknown, for example, whether the introduction of publicly funded tobacco cessation benefits decreases claims for treatment for tobacco-related diseases. In this longitudinal study (a study that follows a group of individuals over a period of time), the researchers ask whether the adoption of comprehensive coverage of tobacco cessation medications by the Massachusetts Medicaid program (MassHealth) in July 2006 has affected claims for treatment for tobacco-related diseases. During its first two and half years, more than 75,000 MassHealth subscribers used the tobacco cessation medication benefit and smoking prevalence among subscribers declined by approximately 10% (38.3% to 28.8%).

**What Did the Researchers Do and Find?:**

The researchers used MassHealth claims data and a statistical method called generalized estimating equations to compare the probability of hospitalization prior to the use of tobacco cessation medication benefit with the probability of hospitalization after benefit use among MassHealth subscribers. After adjusting for other factors that might have affected hospitalization such as influenza outbreaks and the implementation of the Massachusetts Smoke-Free Workplace Law in July 2004, there was a statistically significant annualized decline in hospital admissions for heart attack of 46% after use of the tobacco cessation medication benefit. That is, the calculated annual rate of admissions for heart attacks was 46% lower after use of the benefit than before among MassHealth beneficiaries. There was also a 49% annualized decline in admissions for coronary atherosclerosis, another smoking-related heart disease. There were no significant changes in hospitalization rates for lung diseases (including asthma, pneumonia, and chronic airway obstruction) or for seven other diagnostic groups.

**What Do These Findings Mean?:**

These findings show that, among MassHealth subscribers, the use of a tobacco cessation medication benefit was followed by a significant decrease in claims for hospitalization for heart attack and for coronary atherosclerosis but not for other diseases. It does not, however, show that the reduced claims for hospitalization were associated with a reduction in smoking because smoking cessation was not recorded by MassHealth. Furthermore, it is possible that the people who used the tobacco cessation medication benefit shared other characteristics that reduced their chances of hospitalization for heart disease. For example, people using tobacco cessation medication might have been more likely to adhere to prescription schedules for medications such as statins that would also reduce their risk of heart disease. Finally, these findings might be unique to Massachusetts, so similar studies need to be undertaken in other states. Nevertheless, the results of this study suggest that, for low-income smokers, removing financial barriers to the use of smoking cessation medications has the potential to produce short-term decreases in the use of hospital services that will, hopefully, outweigh the costs of comprehensive tobacco cessation medication benefits.

**Additional Information:**

Please access these Web sites via the online version of this summary at http://dx.doi.org/10.1371/journal.pmed.1000375.

- The US Centers for Disease Control and Prevention Office on Smoking and Health has information on all aspects of smoking and health, including advice on how to quit
- The UK National Health Service Choices Web site provides advice about quitting smoking; more advice on quitting is provided by Smokefree
- The American Heart Association provides information on heart disease, including advice on how to quit smoking (in several languages)
- Information about MassHealth is available, including information on smoking and tobacco use prevention

## Introduction

Cigarette smoking is the leading preventable cause of death in the United States. It also contributes to health disparities, as tobacco use is highest in individuals with less education and lower incomes. In the short term, the only way to decrease tobacco use rates is to increase population-wide smoking cessation rates [1],[2]. This decrease can be achieved by encouraging more smokers to try to quit and/or by increasing the success of those quit attempts with effective treatment, which includes counseling and/or pharmacotherapy with nicotine replacement products, bupropion, or varenicline [2],[3].

At the population level, smoking cessation attempts and quit rates can be increased by reducing the cost of treatment to the smoker [3]–[5]. Smoking cessation treatment is not well covered in current health insurance programs, especially in state Medicaid programs that cover low-income individuals, who are more likely to be smokers. Currently, only 45% of state Medicaid programs offer tobacco cessation treatment that includes both pharmacotherapy and counseling, but only 12% cover behavioral counseling and all medications approved for tobacco cessation treatment by the US Food and Drug Administration (FDA) [6].

A barrier to the adoption of comprehensive tobacco treatment coverage, especially by publicly funded health insurance programs is the projected impact on health care costs. The health care costs of smokers who quit decline within 2 to 5 y after quitting [7]–[10], but the delay in cost recovery has been a barrier to governments considering adoption of smoking cessation benefits. Without better evidence of health improvements or cost containment, it is difficult for policy makers to mandate benefits that will incur significant expenses, especially in light of return-on-investment (ROI) models that show short term increases in health care costs following tobacco cessation. To date, to our knowledge, no US state has examined the impact of a publicly funded tobacco cessation benefit on medical claims for tobacco-related diagnoses.

In 2006, as part of a comprehensive health care reform law, Massachusetts mandated tobacco cessation treatment for all subscribers aged 18 y and older who were insured through MassHealth, the state's Medicaid program. Prior to this law, MassHealth did not provide tobacco cessation benefits to its subscribers. Starting July 1, 2006, the tobacco cessation benefit provided comprehensive coverage for both pharmacotherapy and counseling with minimal copay. On the basis of secondary surveys from the Behavioral Risk Factor Surveillance System (BRFSS), it was estimated that nearly 40% of smokers on MassHealth used the benefit to obtain either prescription or over-the-counter medications to help them quit [5]. In the first 2.5 y after this low-barrier insurance coverage was offered for tobacco cessation medications, a significant drop in smoking prevalence was observed among the Massachusetts Medicaid population [5]. Using BRFSS survey responses, smoking prevalence in the prebenefit period was estimated to be 38.3%. The rate dropped to 28.8% 2.5 y later. Moreover, a joinpoint analysis indicated that the drop in prevalence coincided with the implementation date of the MassHealth tobacco cessation benefit. BRFSS data for this period also showed no change in the percentage of smokers making quit attempts (1 d or longer). However, there was a significant increase in the percentage of former smokers reporting recent quit success. Specifically, the percentage of smokers reporting that they quit smoking in the previous 12 mo rose from 6.6% in the prebenefit period to 19.1% in the postbenefit period. Taken together, these findings suggest that a tobacco cessation benefit with low barriers can significantly reduce smoking prevalence in a Medicaid population.

The present study analyzes MassHealth claims data to explore the effect of comprehensive coverage of smoking cessation treatment on MassHealth subscribers' use of hospital care, which is a major contributor to overall health care costs [11],[12]. Because these claims data do not include information about the smoking status of individuals using health services, we could not compare the claims experience of individuals by smoking status over time. Instead, we compared MassHealth subscribers' rates of hospitalization for specific diagnoses as a function of time before and after use of the tobacco cessation pharmacotherapy benefit, controlling for trends in hospital care utilization.

We hypothesized that a subscriber's probability of hospitalization for tobacco-related diagnoses would decrease as a function of time after use of the tobacco cessation benefit when compared to the same individual's probability of hospitalization prior to the benefit use. We further hypothesized that this postutilization reversal of risk would vary by diagnosis. Tobacco-related diagnoses with more rapid risk reductions would likely show significant reductions in probability of hospitalization, while those diagnoses with longer term risk reductions would not.

## Method

### Study Design

We conducted a longitudinal analysis comparing MassHealth subscribers' rates of hospitalization for specific diagnoses before and after their first use of the tobacco cessation pharmacotherapy benefit, controlling for trends in hospital care utilization. MassHealth and the Massachusetts Department of Public Health (MDPH) operate under the umbrella of the Massachusetts Executive Office of Health and Human Services (EOHHS). All EOHHS employees are required to be trained regarding ethics, confidentiality, and privacy issues related to the use and dissemination of health data. A data sharing agreement for this project was prepared by MassHealth and signed by representatives of MassHealth and MDPH. This agreement required that all claims records be stored on a secure password-protected server. Access to the claims records was limited to four of the authors on this paper (TL, MP, LW, and LK).

### Tobacco Cessation Benefit

The tobacco cessation benefit, which began on July 1, 2006, provided coverage for both pharmacotherapy and counseling. With a doctor's prescription, MassHealth subscribers could obtain FDA-approved smoking cessation pharmacotherapies for US$1– US$3 for a 1-mo supply including over-the-counter medications. No preauthorization was required for the nicotine patch, gum, or lozenge, bupropion, or varenicline. Smokers could obtain a 90-d supply up to twice per year. In-person smoking cessation counseling services were also covered by the benefit. The state already provided up to five sessions of free telephone counseling through the state's quitline; this continued unchanged with the new cessation benefit. The counseling services were not required in order for subscribers to get pharmacotherapy.

### Population

The population consisted of MassHealth subscribers who used the tobacco cessation pharmacotherapy benefit. The analysis was limited to use of the pharmacotherapy benefit, because use of the counseling benefit was very low compared to use of the pharmacotherapy benefit; 97% of all claims were pharmacotherapy claims. Since 2006, this percentage has varied less than 1% year to year. Of all subscribers who used the tobacco benefit, 98% had at least one claim for a tobacco cessation medication.

Use of the benefit was defined as having a claim for a prescription of an FDA-approved tobacco cessation medication (any nicotine replacement product or varenicline). Subscribers who had a claim for a bupropion prescription were excluded from the analysis because the drug is not prescribed only for smoking cessation. To be included in the analysis, recipients were required to have a prescription for a tobacco cessation medication filled between July 1, 2006 and November 17, 2007. The end date was chosen to allow for a minimum of 6 mo of postutilization claims. In addition, recipients had to have at least 321 d of MassHealth eligibility in the 365 d both prior to and after the use of the benefit, excluding days where the recipient was dually eligible for Medicare and Medicaid, and had to be MassHealth eligible for at least 51 d in each 8-wk time segment, excluding days where the recipient was dually eligible for Medicare and Medicaid.

### Data

MassHealth prepared raw eligibility and claims files for all subscribers who used the tobacco cessation benefit prior to November 17, 2007. Claims were included from all three types of MassHealth plans: fee for service (FFS), primary care clinician (PCC), and managed care organization (MCO). Full claims data were available for each plan, including the MCO that operates under a prospective payment scheme. The records included claims for inpatient hospitalizations (e.g., hospital specific charges), outpatient events (e.g., emergency department charges), physician services, medical services (e.g., hospice, physical therapy), and pharmacy prescriptions. All data records included a claim date or a specific date of service. All records except pharmacy claims included up to five International Classification of Diseases, 9th edition Clinical Modification (ICD 9) diagnosis codes.

Data for individual recipients were organized to produce a type of health history. Each history was broken into 33 consecutive 8-wk segments that were designed so that the implementation date of the tobacco cessation benefit (July 1, 2006) was the start of one of the 8-wk segments. The first segment began on August 2, 2003, and the implementation date of the MassHealth tobacco cessation benefit occurred at the start of the twentieth segment.

### Outcomes

The outcomes of interest were claims for inpatient hospitalization with specific diagnoses during a given time segment. Only primary diagnoses were used. Diagnosis groups were defined according to the Healthcare Cost and Utilization Project (HCUP) clinical classification system [13]. With the exception of pregnancy or birth-related hospitalizations, all diagnosis groups with at least 200 hospitalizations were evaluated for changes in the likelihood of hospitalization comparing hospitalization rates prior to and following the first utilization date for tobacco cessation medication. Fifteen diagnosis groups had at least 200 hospitalizations in the time frame studied. Four of the 15 diagnostic groups were related to cardiovascular disease (CVD). Four were respiratory conditions. The remaining seven spanned a variety of conditions that were either known not to be related to tobacco use or were smoking-related but had a risk that would not go down in the short term following smoking cessation (Table 1). Hospitalizations for cancer diagnoses were rare and therefore not evaluated.

**Table 1:** Diagnostic group codes evaluated.

Diagnostic Group Codes	Clinical Group Description Based on HCUP Classifications
**Cardiovascular group codes**	AMI (HCUP = 100)
	Coronary atherosclerosis and other heart disease (HCUP = 101)
	Nonspecific chest pain (HCUP = 102)
	Congestive heart failure (HCUP = 108)
**Respiratory group codes**	Pneumonia except by TB or STD (HCUP = 122)
	COPD and bronchiectasis (HCUP = 127)
	Asthma (HCUP = 128)
	Respiratory failure insufficiency arrest (HCUP = 131)
**Other conditions**	Diabetes mellitus with complications (HCUP = 50)
	Biliary tract disease (HCUP = 149)
	Pancreatic disorders not diabetes (HCUP = 152)
	Skin and subcutaneous skin infections (HCUP = 197)
	Abdominal pain (HCUP = 251)
	Mood disorders (HCUP = 657)
	Schizophrenia and other psychotic disorders (HCUP = 659)

STD, sexually transmitted disease; TB, tuberculosis.

Inpatient hospitalization events were recorded in the following manner. For each individual, any 8-wk segment that included an inpatient hospital admission for a specific HCUP diagnosis group was given a value of 1. All time segments for an individual that did not include an inpatient hospital admission for a specific HCUP diagnosis group were assigned a value of 0. Multiple unique admissions in a single time segment were counted only once. For all analyses, the outcome measure was whether or not a hospitalization occurred in a given time segment. The vast majority of periods with recorded hospitalizations included only one inpatient admission per individual for a given period. For schizophrenia and other psychotic disorders, 19% of periods with admissions had more than one admission in that period; this was the maximum value for all diagnostic groups studied. The minimum level for multiple admissions was found for diagnoses of biliary tract disease with 3% multiple admissions. The remainder of diagnostic groups evaluated had approximately 10% multiple hospitalizations for periods with recorded inpatient admissions.

The 8-wk time segment that included the first use of tobacco cessation medications was excluded from all analyses because smoking cessation attempts are often associated in time with adverse health events. Developing new symptoms or receiving treatment for tobacco-related disease can stimulate a smoker to attempt to quit. Standards for hospital quality developed by the Joint Commission assess provision of smoking cessation advice for smokers hospitalized for acute myocardial infarction (AMI), heart failure, and pneumonia [14]. Including the time segment when treatment began in the model would have overestimated the impact of tobacco cessation treatment.

### Independent Variables

Basic demographic data were ascertained from the eligibility file, including gender, age, race/ethnicity, and English-speaking. We also accounted for comorbid medical diagnoses using two methods. First, each segment was scored for health risk during the previous 336 d (six segments) using the Chronic Illness Disability Payment System (CDPS) [15]. CDPS was developed using diagnoses recorded on Medicaid claims records. It has been used to assess health status and to estimate future payments for individual Medicaid subscribers. Also, the HCUP clinical classification system was used to score health risk. All primary and secondary diagnoses were included. The earliest diagnosis date was recorded for nine HCUP categories: AMI, asthma, congestive heart failure, chronic obstructive pulmonary disease (COPD), diabetes, gastritis and duodenitis, hypertension, lupus or other connective tissue disease, and cerebrovascular disease. Those time segments in the patient health history that predated the diagnosis were assigned a value of 0. Those time segments after and including the diagnosis date were assigned a value of 1. Similar coding was undertaken for previous use of medications for treating hypertension and/or hyperlipidemia.

Given the relationship between influenza cases and coronary heart disease (CHD) [16], we also included weekly counts of “influenza-like cases” as recorded by the Massachusetts Department of Public Health. Finally, since research has shown that smoke-free air laws reduce smoking-related health events especially cardiovascular events, we included an indicator for time segments after the implementation of the Massachusetts Smoke-Free Workplace Law (effective date July 5, 2004).

### Analytic Model

Data were analyzed using generalized estimating equations (GEEs) using a logistic link with hospitalization in each time segment as the dependent variable. Generally, we estimated a trend in hospitalization rates prior to benefit utilization and a change in that trend following use of the tobacco cessation benefit. Our primary goal was estimating the magnitude of this change in trend. The general trend was characterized as time in years since August 2, 2003. The change in trend was recorded as the time in years since a recipient's first use of the tobacco cessation benefit. Our primary goal is estimating the magnitude of this change in trend. Start of tobacco cessation treatment was recorded as the earliest time segment in which an FDA-approved tobacco cessation medication prescription was filled. Because many cardiovascular and respiratory conditions have a seasonal quality, annual and semi-annual sine and cosine terms were also included in the model [17]. We adjusted for correlation within individuals across time, assuming a first-order autoregressive structure. All analyses were conducted using SAS 9.1, PROC GENMOD (SAS Corporation).

## Results

Between July 1, 2006, and May 9, 2009, 74,454 MassHealth individual subscribers used the pharmacotherapy benefit. After applying the exclusion criteria described above, 21,656 were eligible for analysis. 35,765 of the original 74,454 individuals were excluded because they first used the benefit after November, 16, 2007, and completeness of claims data for the postutilization time period for these individuals could not be assured. 18,389 individuals were excluded because these subscribers had insufficient eligibility in the year before first utilizing tobacco cessation medications, insufficient eligibility in the year after first use of tobacco cessation medications, or were dually eligible for Medicare. The average case included in the analysis had claims covering 27.5 8-wk segments with an average of 8.7 segments in the postutilization time period.

Table 2 shows demographic and other comparisons for those individuals included in the analysis and those excluded. In general, individuals included were slightly older and more likely to be female, white, and non-English speaking.

**Table 2:** Comparison of benefit users included in analysis to those excluded from analysis.

Benefit User Characteristics	Included (*n = *21,656)	Excluded (*n = *52,798)
First use of tobacco cessation medications between	7/1/2006–11/16/2007	7/1/2006–5/9/2009
Average age (y)	42.1	41.1
Percent male	30.9	43.9
Percent race/ethnicity = white non-Hispanic	71.8	55.9
Percent race/ethnicity = Black non-Hispanic	7.0	5.4
Percent race/ethnicity = Hispanic	5.2	6.0
Percent race/ethnicity = not listed	15.5	31.5
Percent English spoken	73.2	86.4
Percent days eligible for MassHealth (no dual eligible days included)	90.2	46.9
Percent days not eligible for MassHealth	8.4	34.8
Percent days dually eligible for MassHealth and Medicare	1.4	18.3

Among the 21,656 benefit users in the sample, 8,194 (37.8%) had at least one inpatient hospitalization during the study period. In total, there were 17,084 uniquely dated inpatient hospitalizations in the period studied; 71.1% of these hospitalizations occurred in the prebenefit period. The most common primary diagnosis group was asthma, followed by COPD and pneumonia.

Unadjusted rates of hospitalization were calculated prior to accounting for seasonality, influenza, demographics, previous health risks, and smoke-free air laws. Overall, there was a 7% (95% confidence interval [CI] 3%–10%) annualized increase in the unadjusted rate of hospital admissions from the pre-utilization period to the postutilization period (Table 3). There were also increases in the annualized unadjusted rates of hospital admissions for all primary diagnoses of respiratory conditions, congestive heart failure, abdominal pain, and mood disorders. There was a significant decrease in the annualized unadjusted rate of hospital admissions for a primary diagnosis of coronary atherosclerosis (see Table 3).

**Table 3:** Number of admissions by group, unadjusted change in hospital admissions in pre-utilization period compared to postutilization period with *p*-value and 95% CI, annualized change in inpatient hospital admissions postutilization with *p*-value and 95% CI.

Clinical Group Description	*n* Admissions (Pre and Post)	Unadjusted Change Pre-utilization Versus Postutilization			Adjusted Annualized Change Postutilization		
		Annualized Percent Change in Admissions Pre Versus Post (Unadjusted)	*p*-Value Pre Versus Post (Unadjusted)	95% CI Pre Versus Post (Unadjusted)	Annualized Change in Admissions^a^ (Postutilization)	*p*-Value Annualized Change in Admissions	95% CI Annualized Change in Admissions
**Cardiovascular group codes**							
AMI	239	−8%	*P = *0.54	0.70–1.21	−46%	*P = *0.049	0.30–0.98
Coronary atherosclerosis and other heart disease	337	−28%	*P*<0.01	0.56–0.92	−49%	*P = *0.04205	0.28–0.94
Nonspecific chest pain	559	14%	*P = *0.13	0.96–1.36	−32%	*P = *0.07	0.45–1.03
Congestive heart failure	279	103%	*p*<0.001	1.61–2.57	14%	*P = *0.74	0.54–2.37
**Respiratory group codes**							
Pneumonia except by TB or STD	832	16%	*p*<0.05	1.01–1.34	14%	*P = *0.40	0.82–1.62
COPD and bronchiectasis	912	91%	*p*<0.001	1.68–2.18	21%	*P = *0.39	0.79–1.84
Asthma	938	29%	*p*<0.001	1.13–1.48	−1%	*P = *0.95	0.67–1.46
Respiratory failure insufficiency arrest	260	64%	*p*<0.001	1.29–2.10	−6%	*P = *0.84	0.55–1.64
**Other conditions**							
Diabetes mellitus with complications	462	10%	*P = *0.33	0.90–1.33	−3%	*P = *0.93	0.51–1.92
Biliary tract disease	225	−14%	*P = *0.32	0.65–1.15	−13%	*P = *0.67	0.45–1.68
Pancreatic disorders not diabetes	525	4%	*P = *0.68	0.87–1.25	42%	*P = *0.30	0.73–2.79
Skin and subcutaneous skin infections	655	<1%	*P = *0.96	0.84–1.17	−26%	*P = *0.24	0.45–1.22
Abdominal pain	282	15%	*p*<0.05	1.04–1.69	−18%	*P = *0.46	0.48–1.39
Mood disorders	419	23%	*p*<0.05	0.62–0.96	37%	*P = *0.18	0.77–2.43
Schizophrenia and other psychotic disorders	350	−11%	*P = *0.33	0.71–1.12	42%	*P = *0.31	0.67–3.44
**All hospitalizations**	17,724	7%	*p*<0.001	1.03–1.10	−2%	*P = *0.74	0.90–1.08

a Adjusted for trend, seasonality, influenza like cases, individual demographics, prior diagnoses of specific diseases, prior use of hypertension or cholesterol medication, CDPS health risk score, and the implementation date of the Massachusetts Smoke-Free Workplace Law.

STD, sexually transmitted disease; TB, tuberculosis.

Following adjustments for demographics, prior health risks, seasonality, statewide influenza rates, and the implementation date of the Massachusetts Smoke-Free Workplace Law, trend changes in likelihood of hospitalization during the postutilization period were found for AMI and atherosclerosis. Because hospitalizations are relatively rare in our population (19% annual risk of hospitalization), we interpret our odds ratios as changes in the likelihood of hospitalization. For AMI, there was a 46% annualized decrease (95% CI 2%–70%). For coronary atherosclerosis, the annualized decreased was 49% (95% CI 6%–72%). Likelihood of hospitalization for nonspecific chest pain was lower but this change did not reach significance. No other diagnosis group showed a significant increase or decrease in likelihood of hospitalization in the postutilization period.

Quadratic terms were added to all models to test for nonlinearity in the postutilization period. No diagnosis group showed any significant nonlinearity.

## Discussion

Here we extended previous research in smoking prevalence among MassHealth beneficiaries by examining claims data to explore whether the utilization of a low-barrier benefit for tobacco cessation medications is also associated with a significant reduction in hospital care utilization, specifically inpatient hospital admissions for acute coronary heart disease, a tobacco-related diagnosis that is particularly sensitive to decrease in response to smoking cessation [18],[19]. Our analysis of medical claims for MassHealth subscribers who used tobacco cessation medications paid for by MassHealth showed a significant reduction in inpatient hospital claims for two acute CHD diagnoses (AMI and coronary atherosclerosis) in the postutilization period, compared to the pre-utilization period. The findings were robust and persisted after adjustment for potential confounding factors that included demographics, medical comorbidities, seasonality, statewide influenza rates, and the implementation date of the Massachusetts Smoke-Free Workplace Law. We found a 46% annualized decrease in inpatient claims for AMI and a 49% annualized decreased in hospital claims for coronary atherosclerosis claims in the postutilization period. No significant changes occurred in rates of hospital admissions for other diagnoses, including four respiratory conditions (pneumonia, asthma, COPD, and respiratory failure) and in seven additional diagnostic groups not previously associated with smoking or with short-term health improvements following smoking cessation.

Because return-on-investment (ROI) analyses have often shown short-term increases followed by long-term decreases in health care costs for recent quitters, nonlinear trends were evaluated for all 15 diagnostic groups. No diagnosis group showed any significant nonlinearity.

To date, to our knowledge, no study has linked use of the tobacco cessation benefit with a reduction in claims for tobacco-related diagnoses. However, several recent studies have shown reductions in tobacco-related diagnoses following implementation of smoke-free air laws [20]–[22]. Moreover, the impact of smoke-free air laws appears to increase as a function of time in much the same way that the risk of tobacco-related diagnoses decreases after a smoker quits smoking [18],[19]. Therefore, the longitudinal model we used to study the health impact of the MassHealth tobacco cessation benefit mirrors the models used to evaluate the health effects of smoke-free air laws.

This study has several limitations. First, claims records were used as proxies for health events. Review of clinical charts would have yielded a more sensitive accounting of diagnoses but were impractical given the large volume of individual subscribers. Second, unmeasured confounding is a threat to the study's internal validity. Because subscribers were not randomly assigned to the benefit and there was no concurrent control condition, it is possible that subscribers who chose to use the tobacco benefit were also more likely to adhere to treatment for other CHD risk factors such as hypertension or hyperlipidemia. This behavior could independently reduce their likelihood of hospitalization for CHD. To partially address this issue, our model adjusted for simultaneous use of medications for hypertension and hyperlipidemia. However, claims data alone cannot fully address the issue of adherence to prescription schedules. Third, use of the tobacco benefit is used as a proxy for stopping smoking, because smoking status is not available in claims data; this might lead to misclassification of benefit users who did not quit as quitters, and would have the effect of biasing the results toward the null. Finally, Table 2 shows differences between included and excluded subscribers on the basis of eligibility criteria. These differences could limit the generalizability of results to the entire population of MassHealth subscribers.

Because of these limitations, additional studies in other states are warranted. The authors note that initial studies showing reductions in smoking-related diagnoses following implementation of smoke-free laws were met with skepticism. However, subsequent research has greatly increased confidence in the relationship between smoke-free workplace laws and reductions in smoking-related diagnoses, especially myocardial infarction [23]. Those studies used a similar longitudinal model, but only research from other states will determine whether these new results from Massachusetts reflect a replicable pattern of hospital utilization following the implementation of a comprehensive tobacco treatment benefit.

It is unlikely that our findings would have reached significance without the high utilization rate of the Massachusetts Medicaid tobacco cessation benefit. Nearly 40% of subscribers used the benefit in the first 2.5 y after implementation [5]. This rate was achieved, in part, by heavy promotion of the benefit in Massachusetts during the first 18 mo after implementation. The Massachusetts Medicaid Program and the Massachusetts Tobacco Cessation and Prevention Program (MTCP) formed a close working relationship to promote the benefit. In addition, the FDA approved varenicline as a tobacco cessation medication in May 2006. A media campaign by varenicline's manufacturer promoting the product began in December 2006 and may have increased smokers' interest in obtaining smoking cessation treatment.

As noted previously, this study is the second in a series of studies regarding use the MassHealth tobacco cessation benefit. The first study used secondary survey data from the BRFSS to show a significant reduction in smoking prevalence for the Massachusetts Medicaid population. This reduction coincided with the implementation of the tobacco cessation benefit [5]. The current study has focused on reduced inpatient hospitalization claims for tobacco-related diagnoses. Two more papers are planned for this series. The first will focus on evaluating changes in claims for ambulatory visits for Medicaid subscribers who used the MassHealth tobacco cessation benefit. The second will focus on costs and estimated cost savings. The analytic models required for these latter studies are so substantially different from the one presented here that it is necessary to present the material in separate papers.

In preparing this paper, we sought to find other comparable datasets from Medicaid agencies in other states. Little information, if any, was readily available. While demographics may vary from state to state, there was nothing in our results to indicate that the health benefits from quitting smoking would be significantly different in Massachusetts than from any other state's Medicaid population. However, it is still important to note that our study was conducted in a low socioeconomic status (SES) population. Individuals of low SES are at greater risk of a wide range of health conditions through complex and sometimes poorly understood interactions between physical, social, and behavioral mechanisms [24]–[26]. Though we control for preexisting and comorbid health conditions, we cannot know for sure whether unmeasured factors or complex interactions may limit the generalizability of our findings to other populations. Nonetheless, the results reported here are promising. If replicated across state Medicaid programs, these findings have important implications for reducing costly hospitalizations and improving the health of our nation's poorest residents.

## Abbreviations

AMI: acute myocardial infarction
BRFSS: Behavioral Risk Factor Surveillance System
CHD: coronary heart disease
CI: confidence interval
COPD: chronic obstructive pulmonary disease
FDA: US Food and Drug Administration
HCUP: Healthcare Cost and Utilization Project
